# An Ontological Approach of the Cognitive and Affective Product Experience

**DOI:** 10.3389/fnrgo.2021.602881

**Published:** 2021-02-12

**Authors:** David Ribeiro Tavares, Osiris Canciglieri Junior, Lia Buarque de Macedo Guimarães, Marcelo Rudek

**Affiliations:** ^1^Industrial and Systems Engineering Graduate Program (PPGEPS), Polytechnic School at Pontifical Catholic University of Paraná, Curitiba, Brazil; ^2^Production Engineering Graduate Program (PPGEP), Federal University of Rio Grande do Sul, Porto Alegre, Brazil

**Keywords:** ontological approach, cognitive design, affective design, subjectivity, product experience, black box

## Abstract

The cognitive and affective design aims to attract consumers with products and new products that provide innovative experiences with the intense functional and “cognitive” impact such as ease of use, in addition to “affective” impact as the pleasure of consuming. However, it is difficult to anticipate the consumer's preferences and intentionality, because what happens inside his mind, brain, or subjective experience (wishes, needs, and preferences) is not accessible. This study's objective was to propose an ontological and multidisciplinary approach to the cognitive and affective product experience through an explanation framework and a conceptual model. The model was tested, and the preliminary results indicate that the proposal contributes positively to the advance of the explanation, evaluation and translation of the product experience.

## Introduction

Product design is a research field focused on the best practices in the design and development of products and new products. It is usually aimed at meeting consumers wishes, preferences, and needs. Together with engineering, marketing and psychology, it shares common challenges regarding the relationship between product and consumer, the interaction context, and the user experience (UX). Some approaches are more oriented to the consumer and its preferences, while others focus on the product and its attributes. However, they are concerned with subjective aspects known only to the consumer, who lives the subjective product experience. Consumers have their own goals and consumption choices, which can be oriented both to functionality and ease of use, as well as product pleasure and pleasantness. Alternatively, even, to the cognitive and affective qualities offered by the same product. However, when products of different categories and brands, in different markets, need to offer “affective” pleasure and “cognitive” functionality to consumers, the following questions may arise: (***i) what are the reasons for consumers to choose between one product and another?***; (***ii) are there products that offer a more pleasant and affective experience than others?***; (***iii) are there products that are easier and more understandable and produce greater comfort and confidence in meeting the consumer's needs and preferences?***; and (***iv) is it valid to state that products must satisfy both cognitive and functional needs as well as affective and pleasure needs?***.

Products can be easy to use and at the same time, give pleasure to the consumer. In order to achieve this, designers need to recognize the differences between needs for “functional satisfaction” and needs for “emotional satisfaction,” as stated by Khalid and Helander (2004, 2006). However, there is a problem to explain, evaluate and translate the opinions and responses that belong to the cognitive and affective experience. Their subjectivity is considered a “black box” about which not much is known about (Zhou et al., 2013). The challenges imposed by the problem complexity make it challenging to understand the consumer's most personal needs and preferences.

Nowadays, emotional pleasures have gained fundamental importance to the success of products. The creation of deep connections with consumers, through meaningful associations, are valued for having links with their beliefs, experiences, memories, people, places, or even personal values (Noble and Kumar, 2008; Orth and Thurgood, 2018). The strengthening of these subjective bonds, essentially affective, stimulates pleasure and functionality, leading the consumer to choose between one and the other product. Research areas that take product experience into account seek to develop an understanding of consumer experiences by understanding the result of personal interaction with products. Cognitive and affective processes drive experience and shape intentions and decisions about the product. Therefore, the product experience begins with the psychological effects caused by the interaction of the senses. It includes the degree to which all senses are activated, the meanings and values that are assigned to products, and the feelings and emotions caused the interaction (Schifferstein and Hekker, 2011).

Studies on design and product experience go hand in hand with the common goal of proposing products that can be both easy and enjoyable. They seek to promote functional and “cognitive,” and hedonic and “affective” experiences for consumers. Both areas have the same purpose of understanding the subjective elements of the product experience, relevant to identify the subjective intention. However, in addition to the complexity of understanding subjective experience, they face another challenge: affection and cognition have long been treated in psychology as independent entities (Zajonc, 1980; Zhou et al., 2013), which leads to disagreements about the consumer's behavior in product design teams. The separation of emotion and cognition is a deficiency in psychology and cognitive sciences (Vygotsky, 1962; Khalid and Helander, 2006).

Jordan (1998, 1999, 2000), Desmet (2003), and Norman (1988, 2004) were pioneers in approaching the cognitive and affective product design in greater depth. They were also pioneering in considering the different levels of cognitive, affective, and emotional elements—elements that stimulate perception, provoke reactions and responses, and activate the consumer's subjective psychological processes. Therefore, they produce the experience of interacting with the product. Norman (2004) described affection and cognition as information processing systems. The cognitive system gives meaning to the world, while the affective system is critical to it. Each system influences the other, with cognition providing affection and being affected by it (Ashby et al., 1999; Coates, 2003; Crilly et al., 2004). The challenge lies in understanding the mechanics and functioning of human intellectuality and its preferences.

Explain, evaluate and translate the product experience is one of the significant challenges for the research area in UX design, engineering and product design. Few approaches seek to explain the importance of an application that involves all cognitive and affective systems (Khalid and Helander, 2006; Zhou et al., 2013; Jiao et al., 2017). The reason is the complexity of this “minefield” (Khalid, 2006; Khalid and Helander, 2006), or “black box” (Zhou et al., 2013; Diego-Mas and Alcaide-Marzal, 2016; Jiao et al., 2017), which is shown as one of the main problems in the field of product research. Therefore, the separation between affection and cognition affects the ability of design teams to reach a cohesive agreement on product design. Fukuda (2011) attested that it would be a challenge to measure subjective experience and to identify the mapping relationship between experience and the design elements of a product. It is essential to have an application that involves all cognitive and affective systems.

This study aims to propose an ontological and multidisciplinary approach that contributes to the advancement of studies on the complexity of explaining, evaluating and translating the so-called “black box” of subjective experience or product experience (Schifferstein and Hekker, 2011). For that, theoretical assumptions and constructs resulting from the cognitive and affective design literature were associated with a paradigmatic cognitive ontology. Multidisciplinary components extracted paradigm guided the construction of an explanatory framework of cognitive and affective experience of the product. Thus, it was possible to create a theoretical basis for building a conceptual model of evaluation and translation, preliminarily tested for validation.

## Background

### Cognitive and Affective Product Design

The cognitive and affective product design creates and develops products that enhance the product experience. In order to achieve this, it considers cognitive and affective aspects expressed in the consumers' opinions and responses. Through the translation of opinions and responses, they seek to stimulate the consumer's perception using “cognitive” and “affective” attributes and characteristics. Different perspectives are proposed in this direction, and some trends are common in the research field.

**Figure 1:**
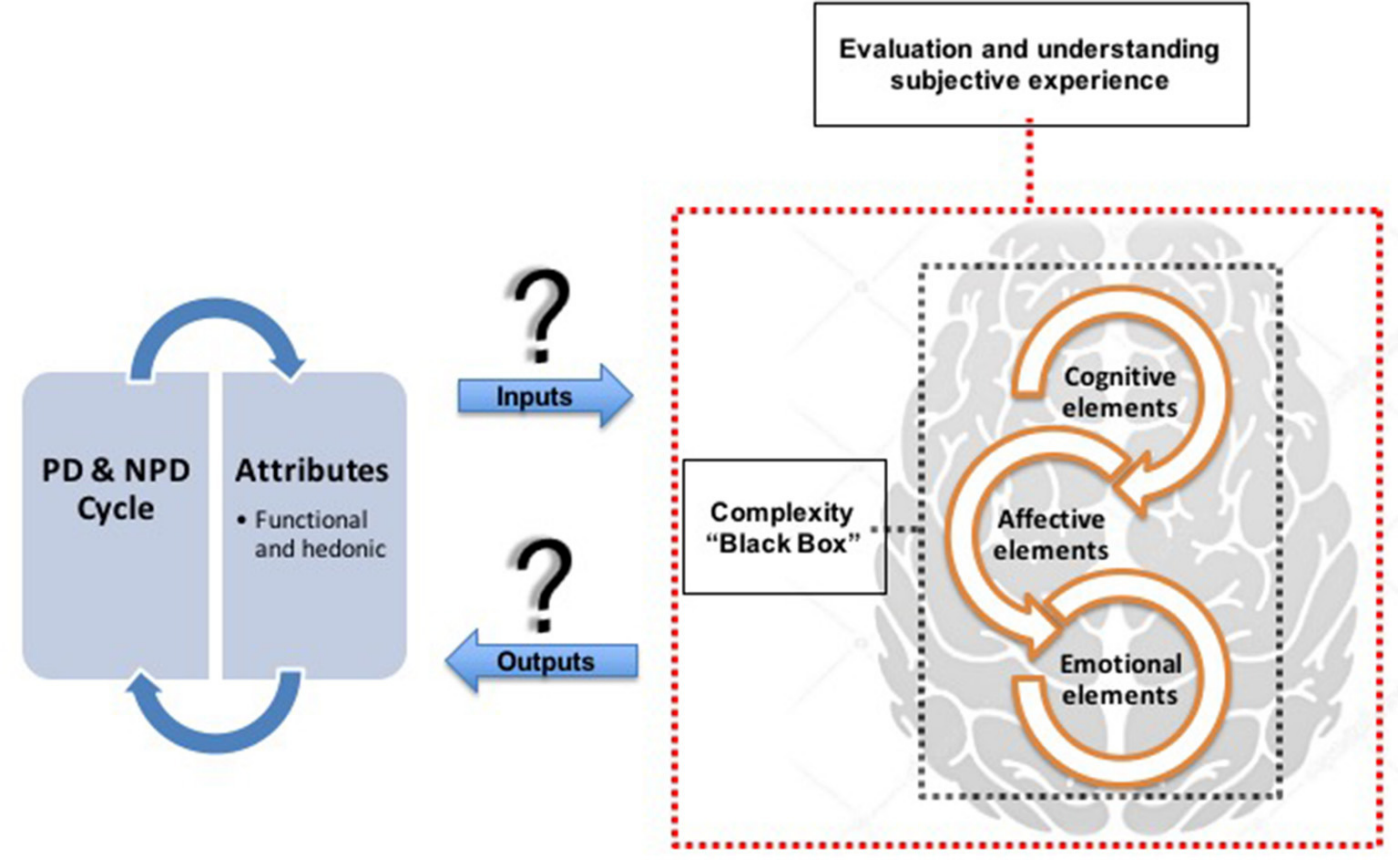
Understanding and evaluation of the subjective experience.

The affective design of products explores the most affective aspects between product and consumer, as proposed by Khalid and Helander (2004, 2006), Khalid (2006), Seva et al. (2011), Diego-Mas and Alcaide-Marzal (2016), and Seva and Helander (2009). The cognitive-emotional design of products proposes a more sentimental, visceral and hedonic approach, as suggested by Crilly et al. (2004), Karim et al. (2017), and Wrigley (2013). Other approaches (e.g., Rindova and Petkova, 2007; Artacho-Ramírez et al., 2008; Li et al., 2014) mixed innovation elements in the cognitive design. There is also the design approach of affective and cognitive experience of products with the user's experience bias (e.g., Zhou et al., 2013; Jiao et al., 2017). These studies share common problems, such as the complexity of understanding and evaluating the subjective experience, or understanding the interaction experience between product and consumer, or even the product experience (Schifferstein and Hekker, 2011), as shown in Figure 1.

### The “Black Box” Complexity of the Subjective Experience of Products

**Figure 2:**
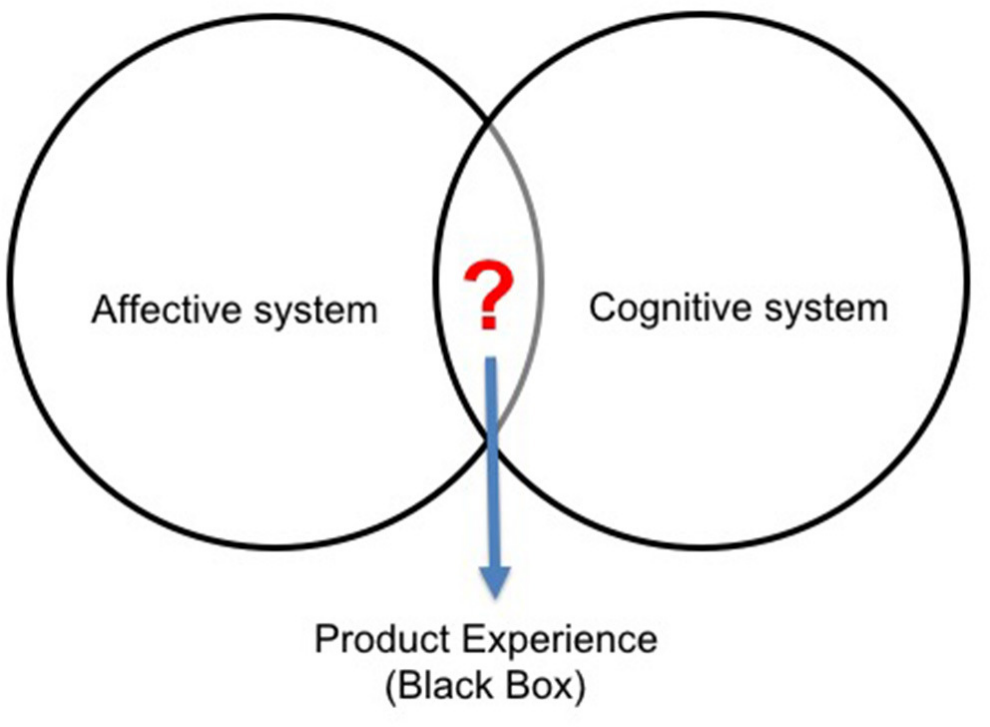
Cognitive and affective systems. Based on Kahneman (2011) and Kahneman and Tversky (1979).

According to Kahneman and Tversky (1979) and Kahneman (2011), two systems essentially comprise the behavior of the human mind: cognitive and affective. In this sense, the information processing performed by these two systems ends up being close to the real subjective experience. It is considered a “black box,” not accessible to engineers, designers and product developers, making it difficult to understand, capture, evaluation and translation of the information correctly, as emphasized in Figure 2. According to Wrigley (2013), emotions can consume up to 80% of an individual's life, while the intellect can control the other 20%. So, emotions directly influence a variety of cognitive responses, and research on emotional effects on product choices is of paramount importance and little explored by product designers and developers (Hirschman and Stern, 1999; Wrigley, 2013).

Several researchers defend the approach of cognition and affection as being a single integrated process. Ellsworth and Scherer (2003), highlighted that while affection refers to sentimental responses, cognition is used to interpret, comprehend and understand the experience. So, cognition must understand and comprehend what is perceived, while affection must promote learning and experiencing the feeling when interacting with the product. Norman (2004) argues that the cognitive system gives meaning to the world, while the affective is fundamental to it. Both complement each other, and each system influences the other, with cognition providing affection and being affected by it (Ashby et al., 1999; Coates, 2003; Crilly et al., 2004). In this sense, Khalid and Helander (2006) affirm that the consumer perceives reality in an affective (intuitive and experiential) and cognitive (analytical and rational) way, and separating emotion from cognition ends up being a significant deficiency of psychology and cognitive sciences. Besides, emotions are not the cause of rational thinking, but they can motivate interest in objectivity. Rational thinking affects feelings, and affective thinking influences cognition, which is why these phenomena are inseparable.

Jiao et al. (2017) attested that the connection between consumer intentions and decisions leads to the recent consensus on affective and cognitive integration in product evaluations. This connection is a complex problem because new methods require different areas of knowledge. The processes that take place until the consumer's judgment, the choice and the factors that affect these decisions vary from one model to another (Engel et al., 1995; Han and Hong, 2003; Crilly et al., 2004; Diego-Mas and Alcaide-Marzal, 2016). Several methods have already been proposed in an attempt to determine the user's response without a verbal evaluation but always with the assumption or simulation of what occurs inside the “black box” (Ho and Lu, 2014; Lu and Petiot, 2014). According to Picard et al. (2004), the cognitive theory designed to explain and explore the role of affect in subjective experience is still in its investigative childhood.

The research that Khalid (2006), Khalid and Helander (2004), and Khalid and Helander (2006) carried out brought significant advances to the understanding of the complexity of the “black box” of the product experience and the processing of the affective and cognitive systems, identified by the authors as fast and slow systems, respectively.

### “Slow” Analytic and “Fast” Experiential Systems

As previously described, there are two systems at work during the product experience: an “analytical” and more cognitive system and another “experiential” and more affective. Epstein (1994) emphasizes that decision-making depends on analytical and cognitive skills that operate slowly. Slovic et al. (2002) considers the system “analytical” or cognitive and uses algorithms and normative rules, such as calculations of probability, formal logic and risk assessment. They have achieved that it is a relatively slow and strenuous system that requires conscious control of the mind. The “experiential” or affective system is fast, intuitive and almost automatic, and does not occur at the conscious level of the consumer. That is, when a person tries to react to an event, there is an automatic search and a match in the memory database, inspired by previous experiences. With it, the human mind searches a history of events past related to the current event in a memory database, including an emotional and as possible of what happened valence. At that moment, mental processing seeks information in the composition of an affective basis for decision making, producing positive or negative emotions.

The challenge is how to understand how products can be designed so that they consider the emotional and experiential processing system to quickly and intuitively information in the definition of their attributes, directly reaching the consumer's affective systems and causing an experience of pleasant consumption.

Therefore, this study finds that affection and cognition are interdependent and must be “integrated” in the product design applications, since the phenomena themselves should be integrated (Parrott and Sabini, 1989; Zhou et al., 2013). Damasio and Adolphs (2001), and Storbeck and Clore (2007) point out that experiments in the laboratory and everyday observation suggest the unity and interrelation of processes trying to separate the negligence possibilities of mental life.

## Ontological and Multidisciplinary Approach to Cognitive and Affective Product Experience

This article discusses the cognitive and affective experience of the product using an ontological approach and multidisciplinary contributions to the advancement of research regarding the “*black box*” of the product experience. So, this study was based on conceptual assumptions and constructs based on the most relevant authors of theoretical advances in the literature. The phenomenon of “black box” will be explained in a new light and from it will be proposed a conceptual model of evaluation and translation of necessary information.

### Assumptions and Conceptual Constructs

The conceptual constructs are presented in the following assumptions: (i) the products have cognitive (functional) and affective (hedonic) attributes; (ii) The “cognitive and affective systems” should be integrated into a single process in product evaluations; (iii) The product experience is the result of a “cognitive and affective” process; and (iv) If there are different “cognitive and affective” requirements.

#### The Products Have Cognitive (Functional) and Affective (Hedonic) Attributes

The first premise establishes that products must have functional and hedonic attributes. Khalid and Helander (2006), Khalid (2006), and Khalid and Helander (2004), who investigated the functional and hedonic aspects (holistic and style), propose “*hedonomy*” as being a new field of research in the design area. These authors believe that the consumer has a subjective experience when interacting with the product. Therefore, the product design can be functional or “*cognitive*” and hedonic or “*affective*” and the answers are opinions about the attributes “*ease of use*,” “*pleasure*,” and “*friendliness*.”

Consumer responses are full of cognitive and affective elements, as they are the result of the integrated functioning of the cognitive and affective systems (Figure 1). The studies carried out by Khalid and Helander (2006) confirm that new products are evaluated preferentially by holistic attributes, and known products are evaluated preferably by attributes of “*style*” and “*functionality*.” The evidence shows that for products that have new features, “*holistic*” and “*style*” attributes are statistically more important than “functional” attributes. Therefore, traditional cognitive design approaches focused only on usability end up underestimating the importance of emotions in product design (Khalid and Helander, 2006).

#### The “Cognitive and Affective Systems” Should Be Integrated Into a Single Process in Product Evaluations

The second assumption is related to the understanding that engineers and designers have about the integration of cognitive and affective systems in product evaluations. According to Norman (1988, 2004), when the consumer analyzes the product, there is a sequence of processes that follows a hierarchy of affective and cognitive levels, and they are described as visceral, behavioral, and reflective levels. Initially, the consumer has an immediate and affective visceral experience when observing the physical appearance of the product and taking pleasure in observing it. Second, the consumer has a behavioral experience when evaluating its usability and performance. Furthermore, finally, the consumer has a more ample reflective experience related to meaning, self-image, and the message conveyed. It is these three *levels* that the authors of this research constitute the cognitive and affective systems in different measures.

Aftab and Rusli (2017) are consistent with the abstraction hierarchy described by Norman (2004) through evaluation experiments in the used furniture design. Most participants are emotionally linked to the usability (behavioral level) and appearance (visceral level) of the evaluated furniture. The constant interaction leads to the realization that the key to a lasting value of the products is in “people who use” and how they give symbolic meaning to objects. It is in that product designer does not provide the meaning, but the users themselves, from their own affective and cognitive experience with the product. Thus, reflective design, the designers propose only the message and consumers interpret the message and give meaning to the emotional significance of the product, i.e., the experience of the product must provide the meaning.

Wrigley (2013) were based on the representation that Crilly et al. (2004) created the *hierarchy* of aesthetic perception, semantic, or functional and symbolic of the product. However, he advanced in research on cognitive and emotional design, incorporating the emotional terminology Norman (2004) on aspects visceral, behavioral, and reflective of product design in the structure developed by Crilly et al. (2004). The processing fulfills two distinct functions in each of the three levels: first, the assessment and judgment of the world and what happens in it (affective) and second, the interpretation of what happens (cognitive). The study suggests a deeper discussion of emotions and their relation to cognition on the paradigm of “emotional cognition,” which includes cognitive and emotional operations in a single product analysis. Although the systems have differences to be considered the same approach to evaluate and influence the other and vice versa.

Thus, cognition understands what the senses perceive sensory, while the affection promotes the feeling and the learning experience during the interaction. This understanding the following proposed concepts Crilly et al. (2004), Khalid and Helander (2006), Norman (1988), and Wrigley (2013). So, cognitive skills interpret the inputs of the product experience and cause emotional memories, evoking associations with other products (Schifferstein and Hekker, 2011).

#### The Product Experience Is the Result of a “Cognitive and Affective” Process

The third assumption is related to the need for greater understanding of subjective consumer experience or interaction experience, or even experience of use, associated with the product experience. Zhou et al. (2013) and Jiao et al. (2017) explored the subjective experience and the affective states that influence the behavior of choice under uncertainty. The authors state that it is possible to improve the consumer experience better understanding their behavior considering all product attributes, integrating cognitive and affective approaches.

There are two important aspects regarding the product experience: first, the internal and subjective experience when using or consuming the product, influenced by the memory of previous learning experiences. There are differences between experienced and inexperienced consumers and, for the first time, Diego-Mas and Alcaide-Marzal (2016) described the product perception process when evaluating the intention to purchase the product, highlighting the importance of the “experience” requirement in the evaluations of products. In this line of thought, Karim et al. (2017) explored the importance of subjective experience in the results of research on the evaluation of consumers' purchase intention. Their research shows that some products produce greater feelings of pleasure compared to others. They confirmed that certain products induce more robust evaluations of pleasure in women than in men. So, it is essential to know how to evaluate the product experience in different cultures and genres in product design.

According to the presented understandings, it is possible to affirm that the affective experience ends up directing the subjective of the evaluation and the consumer ends up associated with the memory of experiences previous to the visceral and aesthetic apprehension of the inputs. From these previous experiences, the consumer can judge whether the product is positive or negative, but still, at pre-reflective, that is affective levels. In this way, reflection is cognitive, requires effort and carries all the subjective affective content. The functioning of this process is internal, subjective, and closed to the external observer, and therefore, it can only be assumed or inferred in empirical analyzes and tests.

#### If There Are Different “Cognitive and Affective” Requirements

The fourth assumption addresses the importance of multi-disciplinary knowledge in order to understand the opinions and consumer responses to the cognitive and affective product design in line of neuroscience and cognitive sciences. According to Maturana and Varela (1987), if the goal is to understand any human activity, then it is necessary to consider the emotion that defines the field of action in which this activity takes place and in the process, learn to observe what actions the emotion you want. Intentions start from subjective emotional and affective internal processes that are expressed. This is essential to understand in depth the phenomenon of subjective experience.

Wrigley (2011, 2013) attested that the response elements of “emotional cognition” are not presented as objective qualities of a product. However, these elements are a cognitive interpretation of the qualities of an object, driven both by the perception of real stimuli and by facts evoked by the consumer's memory and emotion. The response affects the facial muscles and the musculoskeletal structure, the viscera and the internal environment of the body, as well as the neurochemical responses in the brain, and are part of the way in which emotions modify the internal state of the body. Damasio (2001) described it similarly as in its exploitation noted that the instinctive, visceral and immediate response to sensory information strongly influenced the secondary information, acquired when cognitive-behavioral interaction and reflective occurring later. There is a *hierarchy of internal processes in operation*, for although the affection and cognition are, to some extent, different neuroanatomically systems, they are deeply interconnected influencing each other (Ashby et al., 1999; Crilly et al., 2004; Norman, 2004). All are part of the same subjective experience, known in the field of philosophy of mind by the term “qualia,” the result of an internal, subjective experience, inherent in the phenomenon of human “consciousness.” Therefore, a new explanation is needed to elucidate the interconnected functioning of cognitive and affective systems in production of different types of consumers requirements.

### Ontological and Multidisciplinary Components

Cognitive psychology broadens the understanding of the emotional and cognitive phenomenon by attesting that cognition is often the scientific term used to define the thought process. Psychology and cognitive sciences use the term cognition to refer to a view of information processing of an individual's psychological functions. Other interpretations link cognition to the study of all human activities related to knowledge. These activities include attention, creativity, memory, perception, problem-solving, thinking and using language (Wrigley, 2013; Neisser, 2014). Cognition is directly related to emotion, and Norman (2004) defined the domain of emotional cognition as the level of cognitive thinking that deals with emotional responses. For this author, emotional cognition can be seen as emotional processing, experiences, information and day-to-day knowledge encompassing or allowing decision making, action and response.

In the late 1950s and early 1960s, cognitivism replaced behaviorism as the dominant learning theory (Ertmer and Newby, 2013). Pritchard (2014) emphasized the role of “mental” activities in the learning process and including actions such as thinking, remembering, perceiving, interpreting, reasoning and solving problems. This study proposed a theoretical approach to a broad understanding of the human “mind,” recognizing the existence of thought and studying how it affects behavior. Therefore, the act of thinking is not just behavior in itself, but an experience that affects and interferes with the behavior. Unlike behavioral psychologists, cognitive psychologists do not consider that humans are programmed to respond only to environmental stimuli. They believe that individuals can think rationally and learn through active participation, thus creating a subjective experience. This aspect of cognitive theory indicates the importance of understanding human subjectivity.

#### CogPO: Stimulus, Instructions, and Responses

From the cognitive paradigm, studies have accepted and inferred that the types of attributes of a product stimulate consumer perception that, when perceiving and processing inputs, responds with behavioral actions and responses, or outputs. The concept of “stimulus” is used in different scientific fields and, generally, in similar ways. According to Turner and Laird (2012), stimuli are everything that produces a response from an instruction. There are two immediate subtypes of stimuli, indicated and used within the ontology of the cognitive paradigm (CogPO) presented by Turner and Laird (2012): (i) the explicit stimulus; and (ii) the implicit stimulus. The explicit stimulus is usually generated under the control of the experimenter and which exists outside the user, is related to the external stimulus. On the other hand, the implicit stimulus, which is processed internally by the cognitive and affective systems during the subjective experience, is related to the internal stimulus.

**Figure 3:**
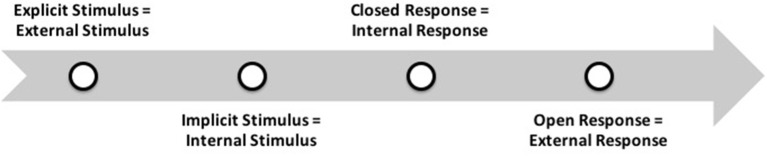
Sequence of cognitive events explained by CogPO. Adapted from CogPO, presented in Turner and Laird (2012).

CogPO was developed from the taxonomy created for the BrainMap database (http://brainmap.org/). This taxonomy seeks to represent the stimulus, instructions and answers that define experimental conditions in a standardized and flexible format, using terms and well-defined relationships. It is one of several ontologies related to knowledge engineering and information integration efforts that work together to think about the relationship between the structure and function of the brain. The instructions provide the entity with information that establishes the rules for the desired behavior of human individuals, i.e., their answers. Indeed, it is an explicit direction, guiding the behavior during the experimental conditions, as shown in Figure 3. The figure shows the logical sequence of events that must be considered from the understanding of CogPO.

The CogPO wiki (www.wiki.cogpo.org) allows a complete view of the process that organizes the two cognitive and affective systems from a cognitive perspective. According to CogPO, a response can be an open response, or a private response performed internally (see Figure 3). The modality that each external or internal response has is the body part or expression used in the response, as well as the opinion of the experiment participant. This article considers the internal responses (subjective experience, domestic and initially unspoken) and external responses (verbalized or marked answers, opinions, expressions and actions) as consumer response requirements. The requirements are essential for the team of engineering and product design evaluate and translate as attributes departing necessarily the subjective experience of the consumer. The concepts and structure presented by CogPO assumed as a fundamental assumption, are adequate and systematic in developing the scope of this study, improving the explanation, evaluation and translation of the cognitive and affective experience of the product.

### Ontological Explanation of the Cognitive and Affective Product Experience

The study aims, as a contribution to the research, to adapt the essentially subjective experience of the product to a cognitive paradigmatic ontology. According to Turner and Laird (2012) this ontology considers: (i) the inputs as stimuli of the product's attributes; (ii) instructions as questions for any experiment carried out and directed to psychological cognitive and affective processes; (iii) outputs such as consumer responses or response requirements. With the assumptions, conceptual and multidisciplinary components construct elaborate and appropriate through CogPO (Table 1), the study seeks conceptually innovating the explanation, evaluation and translation of the cognitive and affective experience of the product, thus bringing new response requirements for the development of engineering and product design team in the process of product development.

**Table 1:** Assumptions, conceptual constructs, and multidisciplinary components.

**Conceptual Constructs**	**Multidisciplinary components**
*i. Products have cognitive (functional) and affective (hedonic) attributes**Assumptions*****:** the products must offer hedonic and functional attributes; the products must be easy and pleasant; the ease of understanding causes positive emotions, while the difficulty causes negative emotions	***External Stimulus (1):*** Inputs originated in the product, more specifically in the attributes
*ii. “Cognitive and affective systems” should be integrated into a single process in product evaluations**Assumptions*****:** cognition and emotion are interlinked in the perceiving process of a product; cognition interprets and understands, while affection judges experience; the cognitive and affective systems are integrated in subjective analysis and decision-making	***Internal Stimulus (2):*** Sensory perception detects the stimulus originated in the product through instruction (question). Sensory and cognitive perception initiates the slow and analytical processing, the product interpretation and understanding that is overlaid by the fast and intuitive affective system, a faster system, which lives the experience
*iii. The product experience is the result of a “cognitive and affective” process**Assumptions*****:** the affective and automatic system dominates the cognitive and thought system; affective heuristic biases are faster and more intuitive than rational cognitive decisions, which are slower and more analytical	***Internal Response (3):*** This moment defines how the subjective experience that occurs from heuristic biases based on memories, beliefs, affections and emotions will be. It determines whether the product experience will be positive or negative. The processes unfold into subjective visceral and aesthetic (affective), behavioral and semantic (cognitive), symbolic and reflective (affective and cognitive) experiences. It is accepted that they affect the consumer's purchase intention by performing automatic associations and comparisons in the memory bank of previous experiences
*iv. If there are different “cognitive and affective” requirements**Assumptions*****:** emotional responses are predictors of purchase intent; the cognitive responses have elements of aesthetic impression, semantic interpretation and symbolic association; the cognitive requirements can be classified into visceral, behavioral and reflective; the affective and emotional (hedonic) needs and preferences operate as decision-making heuristics and direct the intentional bias; it is possible to identify the consumer's subjective perception	***External Response (4):*** The result is external responses considered as the consumer's response requirements. The requirements follow the CogPo understanding and, as a research proposal, they can represent indicators such as cognitiveness (ease or difficulty), affectivity (pleasantness or unpleasantness) and intentionality of purchasing the product, which influences the purchase decision

The stimuli (product attributes) are perceived and evaluated by consumers through instructions (questions) directed to their opinions (answers). Instructions (questions) are directed to the eight levels of subjective experience accepted as response variables, as shown in Table 2. The authors believe that the eight experience levels contribute to the explanation of consumer experience with the product through the targeting systems, affective and cognitive dimensions and also the cognitive and affective product attributes.

**Table 2:** Instructions (questions) directed to specific cognitive and affective systems/dimensions.

**System**	**Subjective experience level**	**Instruction-question about appearance of usability and style**
Affective (Fast)	Visceral (sensation)	Q.1: What “feelings” do you have when watching this product?
	Aesthetics (aesthetic perception)	Q.2: “Aesthetically” speaking, is this product unpleasant or pleasant for you?
	Symbolic* (association of images with affective meaning)	Q.5: Does the style of this product have an unpleasant or pleasant “meaning” for you?
	Reflective* (affective reflection)	Q.6: After thinking and “reflecting” a little about this product style, do you consider it unpleasant or pleasant?
Cognitive (Slow)	Behavioral (usability)	Q.3: What is your “perception” about the “ease of use” of this product?
	Semantics (message, communication)	Q.4: Can you “understand” the “functions” that this product proposes
	Symbolic* (association of images, cognitive meaning)	Q.7: Does this product's ease of use have a difficult or easy “meaning” for you?
	Reflective* (cognitive reflection)	Q.8: After thinking and “reflecting” a little about this product's functionality, do you consider it difficult or easy?
Variable Intention	Intention bias (confirmatory)	Q.9: Would you buy this product?

As response requirements, the study explored in this research identifies the opinions derived from the eight levels of subjective experience suggested (Table 2): (i) “affective” (visceral and aesthetic); (ii) “cognitive” (behavioral and semantic); and (iii) “affective-cognitive” (symbolic and reflective) concerning “functional” and “hedonic” product design. The objective is to unite the “cognitive and affective” aspects of the consumer with the “cognitive and affective” design of the product, represented in a context of product experience that unites the two parts. A scale from 0 (difficult/unpleasant) to 10 (easy/pleasant) accompanies all questions. The verification of “cognitive” ease is related to the functional attribute (i.e., usability of appearance), and the verification of “affective” pleasantness is related to the hedonic attribute (i.e., style). Through the eight response variables resulting from the instructions, it is possible to evaluate the functional and “cognitive” attributes of the product using the *cognitiveness indicator* and the hedonic and “affective” attributes of the product using the *affectivity indicator*. Besides, by averaging these two indicators, it is possible to assess *intentionality*, which is an important indicator for product design and engineering teams to identify the consumer's subjective intentionality.

Table 3 highlights the indicators that result in cognitive and affective experience of the product. Responses may be low cognitiveness (CRs < 5) or high (CRs > 5), which indicates a product that is difficult or easy to consume in customer perception. Indicators can also be low (CRs < 5) or high (CRs > 5) affectivity, which indicates an unpleasant or pleasant to consume the product in customer perception. When calculating the average of the two indicators, it is possible to obtain the intentionality indicator (the average of cognitiveness and affectivity), it can be of weak (CRs < 5) or strong (CRs > 5). Instruction 9 (Table 2) allows the intention of providing a validation indicator variable “purchase intent” and serving as the base and to analyze the correlation with the intentionality indicator.

**Table 3:** Consumer requirements (CR's).

Consumer requirements (CR's)	1) Affectivity indicator (response variables for Q.1, Q.2, Q.5 and Q.6)	0 to 10	<5 <	Low/High
	2) Cognitiveness indicator (response variables for Q.3, Q.4, Q.7 and Q.8)	0 to 10	<5 <	Low/High
	3) Intentionality indicator (average of 1 and 2)	0 to 10	<5 <	Weak/Strong

*Bold values indicates the measure of the indicator, whether low or high, weak or strong*.

**Figure 4:**
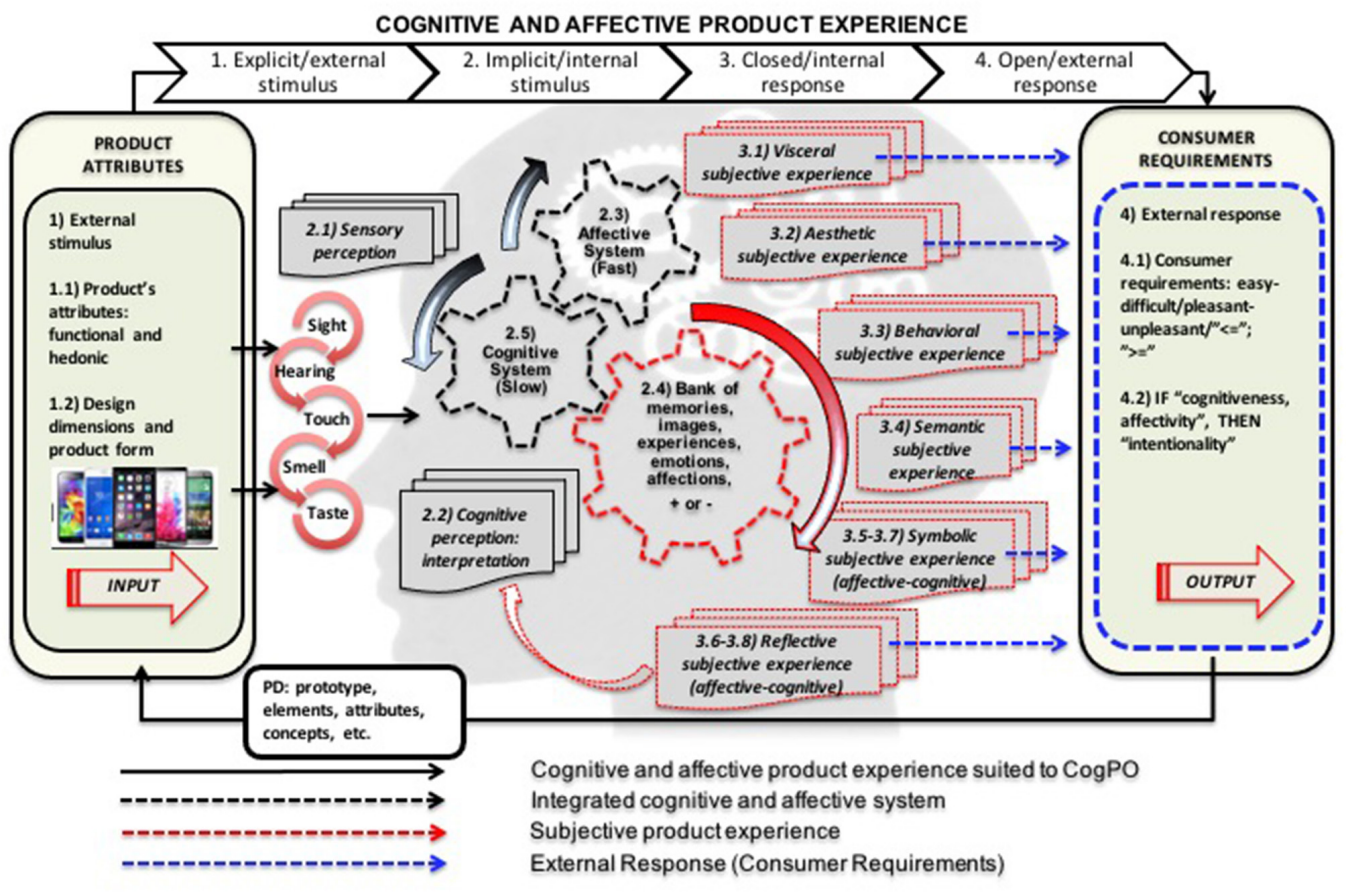
Framework for the explanation of the cognitive and affective product experience.

Through the conceptual constructs associated with the multidisciplinary components (CogPO), the instructions and responses in the form of indicators, it was possible to design a conceptual framework capable of explaining the cognitive and affective experience of the product. The explanation structure will be used as a basis for the process of evaluating and translating the product experience. Figure 4 describes this conceptual framework for understanding the product's cognitive and affective experience. This framework is based on the *hierarchy levels* of the cognitive and affective systems according to the issues obtained from the studied literature (Crilly et al., 2004; Khalid and Helander, 2004; Norman, 2004; Wrigley, 2011; Zhou et al., 2013). The challenge is to understand how different consumer groups operate the process hierarchy. The framework respects the hierarchy of cognitive and affective systems and incorporates the ontology of the cognitive paradigm to systematize and order a new explanation of the “black box” phenomenon. The explanatory framework is composed of: (i) external stimuli (1), identified as inputs caused by product attributes (1.1, 1.2); (ii) internal stimuli (2), identified with the process of perceiving the inputs (2.1, 2.2) and understanding the product through the consumer's cognitive and affective systems (2.3, 2.4, 2.5); (iii) internal responses (3), identified as visceral (3.1), aesthetic (3.2), behavioral (3.3), semantic (3.4), symbolic (3.5–3.7) and reflective (3.6–3.8), subjective experiences.

The explanatory framework suggests that the sum of the subjective experience levels (3.1–3.8) integrates the product experience and is the result of the processing of inputs performed by the cognitive and affective systems in an integrated manner. The answers are the result of instructions-questions directed to the specific processes of each level of experience.

Therefore, external responses (4) are the outputs identified and associated as the consumer response requirements (4.1). Therefore, framework proposes these requirements in the form of three indicators (4.2): (i) cognitiveness indicator, which measures the cognitive or functional aspects of the product, (ii) affectivity indicator, which measures the affective or pleasant and pleasure aspects of the product; and (iii) intentionality indicator, which measures the relationship between cognitive and affective indicators. The intentionality indicator reveals the consumer's intention as a result of their cognitive and affective experience with the product and will be confronted with question 9 (Tables 2, 3).

With the information presented, product design teams can focus their efforts on the type of product attribute that needs to be improved, whether the product should be more “easy and functional” and therefore more cognitive, or whether the product should be more “pleasant and hedonic,” and therefore more affective. Furthermore, with that, it will be possible to understand better the intentionality of consumers using quantitative measures. The authors believe that the proposed conceptual framework can be applied in the areas of marketing and advertising, as well as in psychological studies on the relationship of use and consumption existing in the context of product experience.

### Validation

Preliminary validation of the conceptual framework was performed by six professional engineering and product design based on instructions (issues) in Table 3 (Questions directed to specific cognitive and affective processes or tasks). Suggestions for improvements as well as the description of the implementations were taken into consideration the consumer response requirements presented in Table 4. With the suggestions, a conceptual model for evaluating and translating opinions and responses was defined that crosses the “cognitive and affective” inputs of the product (stimuli) with the “cognitive and affective” outputs of the consumer (responses), through the questions (instructions) directed to different levels of product experience, as suggested by Figure 4.

**Table 4:** Suggestions and improvements to the explanation framework.

a) Inversion of measurement scales for functional and “cognitive” and hedonic and “affective” attributes	- For cognitive aspects, measuring opinions on scales from difficult (1) to easy (10); and of affective aspects, the measurement of opinions in scales from unpleasant (1) to pleasant (10)
b) Notes on visceral and aesthetic aspects are the same for average user	- We chose to keep the different questions in order to seek to evaluate the subjective experience with a greater degree of breadth, considering the hierarchies proposed by Crilly et al. (2004), Khalid and Helander (2004), Wrigley (2013)
c) Notes on the aesthetic aspect interfering with the functional aspect of the product	- Important information for the validation of the proposed model that considers aspects at different levels of assessment of consumer's cognitive and affective experience
d) Clearer separation of questions that are directed to the functional and more “cognitive” and aesthetic and more “affective” aspects of the products	- Checked in the association, as proposed in the model, of functional attributes to be measured by cognitive scales (difficult to easy), and aesthetic attributes by affective scales (unpleasant to pleasant)
e) Concern of users during application of the questionnaire with their previous answers	- The text has been adjusted and the question size has been reduced
f) Semantic proximity to some questions	- Solved with application of different measurement scales for separate assessment of the cognitive and affective dimensions
g) Mix the order of the products during the experiments	- To be considered in applications.
h) Increase the size of images	- To be considered in applications
i) Users start to compare products	- To be considered in applications
j) Mude as questões durante o experimento	- To be considered in applications
k) Measure perception of ease of use	- Directed the translation of functional requirements of the products
l) Ask if the user has the product	- To be considered and associated with the usage experience requirement
m) Cross with the best-selling products	- To be considered in applications
n) Use easy products, such as household appliances	- To be considered in applications

## Conceptual Model of Evaluation and Translation

**Figure 5:**
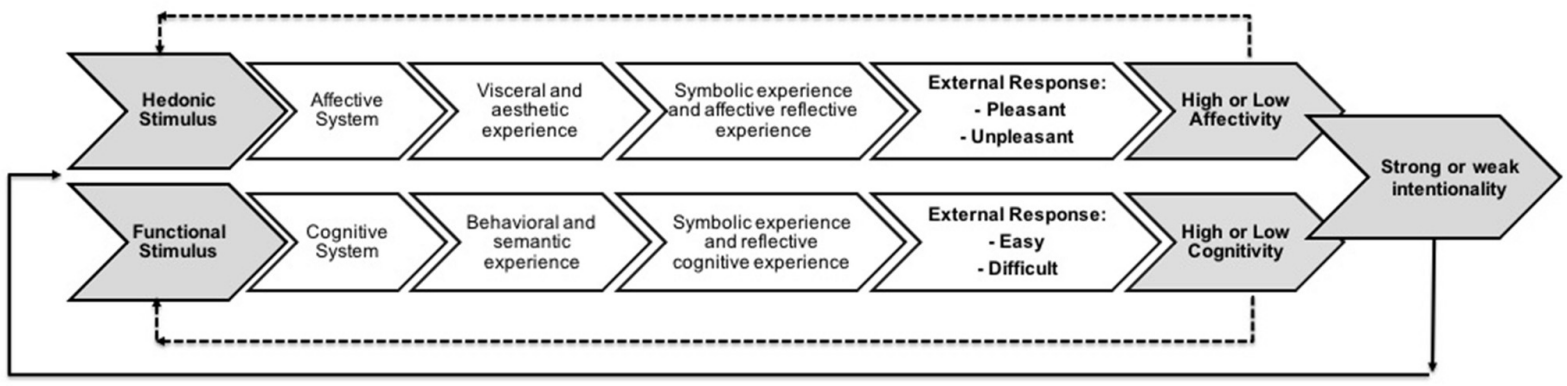
Conceptual model of evaluation and translation.

Figure 4 is the basis for the conceptual model. The consumer response requirements are the result of the cognitive and affective product experience and expressed in opinions regarding the instructions-questions aimed at functional and “cognitive” as well as hedonic and “affective” attributes of the product. Opinions contain cognitive and affective elements and are the result of the subjective experience, visceral and aesthetic, behavioral and semantic, symbolic and reflective, presented in the form of indicators, as shown in Figure 5.

The conceptual model of evaluation and translation is based on the following assumptions. First, it assumes that the hedonic attribute or stimulus demands the affective system and produces a response regarding the pleasantness or dislike of using or consuming the product. It is the result of visceral and aesthetic affective experience, later symbolic and reflective. It represents the indicator of affectivity. Second, is that the attribute or functional stimulus demands the cognitive system and produces a response regarding the ease or difficulty of using or consuming the product. It is the result of cognitive-behavioral and semantic experience, later symbolic and reflective, and represents the indicator of cognitiveness.

The experiences, both affective and cognitive, are followed by the subjective symbolic and reflective experience, as they are composed of superior processes of the “human mind” and belong to late hierarchical levels (Norman, 1988; Crilly et al., 2004; Seva et al., 2011; Wrigley, 2013). Therefore, the authors assume that the subjective symbolic and reflective experience is found at the end of the process of judging and discerning the product experience. Therefore, they are related to both the hedonic attributes and the functional attributes of the product. So, the totality of affectivity and cognitiveness measures are considered in the formation of the consumer's intentionality.

### Preliminary Study

A preliminary study was carried out to validate the model. The application objective is to verify whether the variables (Q1 to Q8) are within the suggested cognitive and affective dimensions, and whether the resulting cognitive and affective indicators are valid to build the consumer's intentionality indicator in relation to the product. The results can confirm the assumptions that have affective dimensions of variables strongly correlated with affective attributes and variables of cognitive dimensions are strongly correlated with cognitive attributes. Also, if the suggested cognitive and affective indicators may influence the intention to purchase by the consumer.

#### Survey

The questionnaire (Table 2) was made available on the web through Google Forms platform. The link was sent via e-mail and other channels to over a thousand participants and was to return 111 validated questionnaires for data analysis. Using a 11-point scale (0 to 10), called “Phrase Completion Scale” (Hodge and Gillespie, 2003), participants answered the 9 questions for each product separately.

#### Test Products

**Figure 6:**
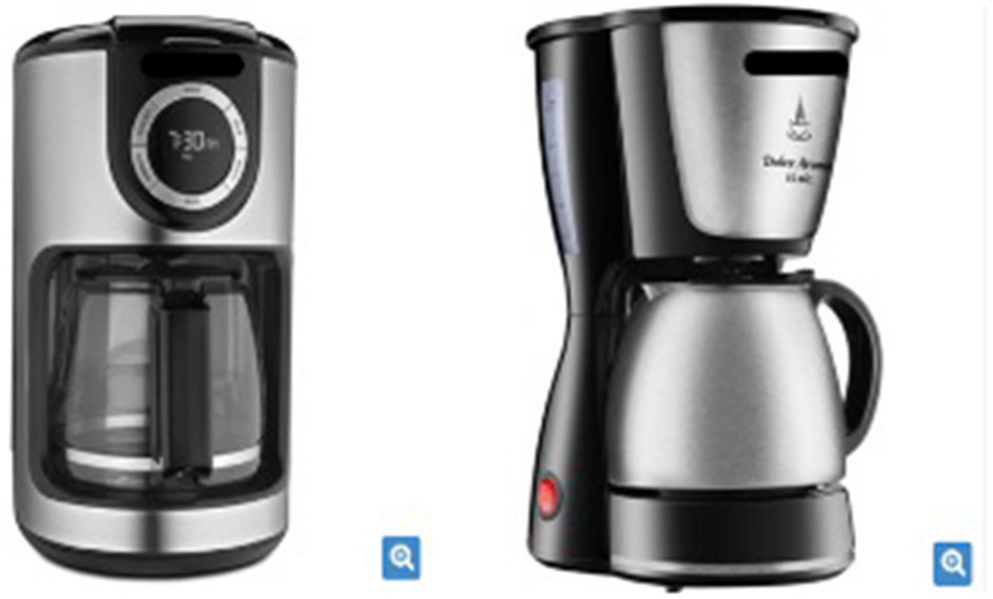
Digital and analog coffee makers.

The study chose a popular consumer product (Figure 6)—a digital coffee maker (left side) and an analog coffee maker (right side). The product types were chosen for their relevance and regional characteristic of experiment application (South region of Brazil), where the majority of the local population uses the product or a similar one.

As proposed in the model, two attributes were chosen to assess the cognitive and affective experience of the product. The functional and “most cognitive” attribute chosen for the consumer assessment was the “usability appearance” of the product, the hedonic and “most affective” attribute chosen for the consumer assessment of the consumer was the “design style” of the product, considering the research developed by Seva et al. (2011) and Rindova and Petkova (2007), respectively. At this point, it should be noted that what was sought to demonstrate was the validity of the model and its correlations, and not the specific opinion of consumers concerning the products, being this object of future exploration.

### Discussion and Results

The study applied an exploratory factor analysis (FA) using IBM SPSS software for data analysis. The objective was to verify the cognitive and affective dimensions and its relations with the eight proposed variables and build from them indicators able to measure the user experience with the product. To check the internal consistency of the nine response variables were calculated using Cronbach's alpha (Cronbach, 1951). The coefficients generated for each construct or dimension as shown in Table 5. The Cronbach's alpha found is more significant than 0.9, which indicated adequate internal consistency for the dimensions and measures could be grouped by items that compose (Landis and Koch, 1977).

**Table 5:** Results of conceptual model application.

**Dimension**	**Variable**	**Experience Level**	**Factor Loading**
Cognitive EV = 41.21% (Cronbach's Alpha 0.928)	**X1**	Reflective Cognitive	**0.9315**
	**X2**	Symbolic Cognitive	**0.9270**
	**X3**	Semantic	0.8688
	**X4**	Behavioral	0.8396
Affective EV = 40.53% (Cronbach's Alpha 0.915)	**X5**	Affective Reflective	**0.9049**
	**X6**	Affective Symbolic	0.8777
	**X7**	Aesthetic	0.8777
	**X8**	Visceral	0.8552
Total Explained Variance (EV) =			**81.74**

*Bold values indicates most relevant factor loads, as well as the explained variance*.

The factorial loads indicated correlations between the original variables and the dimensions of the study, i.e., the more significant the factor load, the greater the correlation with a given dimension. In this sense, the cognitive dimension was 41.21% of the explained variance (EV), while the affective dimension was 40.53% of the EV. The total explained variance was 81.74%, which is higher than the recommended variance (EV > 0.7 or 70%). The results confirmed the affective variables in the affective dimension (X5 to X8) and the cognitive variables in the cognitive dimension (X1 to X4) with high factor loads. Therefore, the results confirm the two initial premises. The results also confirmed that the symbolic and reflective variables could be affective and cognitive (since the symbolic and reflective experience followed both). The symbolic variables also belong to late hierarchical levels that are at the end of the judgment and insight process in the product experience and confirmed by the highest factor loads found (see Table 5). It means that these variables have a stronger correlation with both the cognitive and affective dimensions. Through the dimensions and factorial loads of the explained variables, it was possible to define equations that make up the indicators of cognitiveness, affectivity and intentionality, as described in detail in Table 6.

**Table 6:** Cognitiveness, affectivity and intentionality indicators.

Cognitiveness Indicator =	(X1*0.9315 +X2*0.9270 + X3*0.8688 + X4*0.8396)/4
Affectivity Indicator =	(X5*0.9049 + X6*0.8777 + X7*0.8777 + X8*0.8552)/4
Intentionality Indicator =	(Cognitiveness Indicator + Affectivity Indicator)/2

**Figure 7:**
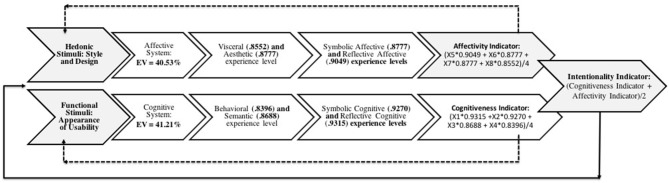
Results of applying the conceptual model.

The indicators of cognitiveness and affectivity resulting from the levels of subjective experience are perceived as a strong measure for the evaluation of consumer's perception, in relation to the product's different functional and hedonic attributes, that is, to translate his/her product's experience. From the high factor loads, that indicate strong correlation, it is understood that the intentionality indicator can be a strong measure to identify the consumer's purchase intention and should be tested in future research for its validation. Authors suggest that the validation of indicators in future researches are found the correlation between the indicators of cognition and affectivity with the indicator of intention. As noted in the previous section, the study included one last question to check purchase intent (see Table 2, Instruction Q.9). The latter response variable ends up being the result of the question-statement on the intention to purchase or not the product, checking and confirming indicators cognition, affection and intentionality. Based on the intentionality indicator (suggested as an average result of affective and cognitive indicators), the study suggests the possibility of associating the user intent by analyzing the correlations between the eight consumer response variables with cognitive and affective dimensions integrating the product experience, a fundamental premise of the study. Figure 7 summarizes the results of the factorial analysis. The answers of the respondents show a high variance explained for the cognitive and affective dimensions, and high factor loadings that correlate with specific variables (levels of experience), as predicted by theoretical assumptions of the model. Therefore, indicates the strength of cognitiveness and affectivity indicators in the assessment of cognitive and affective experience of the product. In future studies, the data must be processed in order to verify the valuation of products for different consumer groups, as well as confirm the relationship of the two indicators with the intention indicator. At this point, the study focused only validate the conceptual model.

## Conclusion

The research reported in this article was intended to propose an ontological conceptual approach and multidisciplinary to be able to explain, evaluate and translate the cognitive and affective experience with consumer RELAC product. It could represent him with the proposition of an explanatory framework and conceptual model for evaluation and translation. After suggestions for improvements by experts, a preliminary test was used to evaluate the model. The high factor loadings indicated strong correlations between variables suggested as levels of experience and the cognitive and affective evaluated.

The preliminary results obtained were promising and mainly confirmed the theoretical expectations found in the literature. The high factor loads of the response variables that represent the levels of subjective reflexive and symbolic experience (which are proposed in both dimensions) were superior to explain the total variance. They are representative so that the explanation of the subjective cognitive and affective experience occurs in an integrated way, in a unique psychological process. They are also the levels of subjective consumer experience, understood as higher processes of the “human mind,” and belong to later hierarchical levels (Norman, 1988; Crilly et al., 2004; Seva et al., 2011; Wrigley, 2013). In this way, at the end of the affective and cognitive judgment and discernment process, they permeate the “conscious mind” and the subjective qualities of the individual (Tavares, 2018), which means that they are related to each other and must be considered together in the evaluations. Therefore, it is possible to state that, with the highly explained variance of the cognitive (41.21%) and affective (40.53%) dimensions, respectively, the cognitive and affective systems work in line and belong to the same psychological process.

The results indicate that it is counterproductive for the engineering and product design teams to direct their efforts only to the affective and emotional experience or only to the consumer's cognitive experience (Norman, 1988; Crilly et al., 2004; Khalid and Helander, 2004, 2006; Rindova and Petkova, 2007; Artacho-Ramírez et al., 2008; Seva et al., 2011; Wrigley, 2013; Zhou et al., 2013; Li et al., 2014; Jiao et al., 2017). It also indicates that it is counterproductive to direct product design and development efforts only to a specific type of product feature or attribute, without taking into account the different categories of functional and hedonic attributes involved (Khalid and Helander, 2004, 2006; Khalid, 2006).

Cognitive and affective product design seeks to follow this path and narrows the distance between the product and the consumer. This is the research area that seeks to understand the user's subjective experience with the product. In fact, it is the awareness of the psychological effects including the degree to which all the senses are stimulated, the meanings and values attributed to the products and the feelings and emotions that are provoked, according to the research developed by Schifferstein and Hekker (2011). However, the easy and pleasant experience of the product is subjective and varies from consumer to consumer. In this way, opinions and responses result from internal changes in the affective and cognitive systems, inherent to the subjective experience itself, and considered a complex “black box” of understanding and evaluation.

It was possible to demonstrate the importance of considering the affective and cognitive systems/dimensions in an integrated way when applied in the design and product experience assessment. It is possible to verify that affection is decisive for the subjective quality of the product experience and that the cognition is fundamental for the consumer's intention and final decision of choice. The symbolic and reflective, affective, and cognitive processes tend to modify the intensity of a product assessment and should be considered.

The proposed conceptual model for evaluation and translation has a promising potential to address the issues identified in the literature. Therefore, it is not interesting to focus efforts only on assessing the consumer's cognitive or affective systems. Likewise, it is not interesting to direct efforts toward just one type of functional or hedonic attribute of the product, without taking into account the different dimensions that make up a product and the different psychological aspects that integrate the consumer's mind, in addition to the context in which experience happens. For a good user experience with the product, a product must be, at the same time, “cognitive” and “easy,” “affective,” and “pleasant” to use and consume, and this study proposes that this can be achieved with the proposed ontological approach.

In the continuation of this research, the authors will correlate the indicators of cognitiveness, affectivity and intentionality in a broader treatment of the data presented by this study, and in other experiments. In this sense, this study suggests that the framework and the conceptual model be tested, exploring all sensory senses (sight, touch, taste, hearing, and smell), as well as the different types of digital and technological products in different countries and cultures.

## Author Contributions

All authors contributed equally to this work, discussed the reviewed implications, and commented on the manuscript at all stages.

## Conflict of Interest

The authors declare that the research was conducted in the absence of any commercial or financial relationships that could be construed as a potential conflict of interest.
